# Intra-Articular Injection of *Fructus Ligustri Lucidi* Extract Attenuates Pain Behavior and Cartilage Degeneration in Mono-Iodoacetate Induced Osteoarthritic Rats

**DOI:** 10.3389/fphar.2018.01360

**Published:** 2018-11-23

**Authors:** Bo Yan, Li Zhou, Caiwei Wang, Rongrong Wang, Li Yan, Lingying Yu, Fucun Liu, Wenxi Du, Guangping Yu, Qiang Yuan, Peijian Tong, Letian Shan, Thomas Efferth

**Affiliations:** ^1^The First Affiliated Hospital, Zhejiang Chinese Medical University, Hangzhou, China; ^2^Department of Orthopaedics, Changzheng Hospital, Second Military Medical University, Shanghai, China; ^3^Xianju Traditional Chinese Medicine, Taizhou, China; ^4^Department of Pharmaceutical Biology, Institute of Pharmacy and Biochemistry, Johannes Gutenberg University, Mainz, Germany

**Keywords:** *Fructus Ligustri Lucidi*, intra-articular, chondrocyte, hypertrophy, mono-iodoacetate

## Abstract

*Fructus Ligustri Lucidi* (FLL) has been widely used as a traditional Chinese medicine (TCM) for treating soreness and weakness of waist and knees. It has potential for treating OA owing to its kidney-tonifying activity with bone-strengthening effects, but there is so far no report of its anti-OA effect. This study established a rat OA model by intra-articular (IA) injection of mono-iodoacetate (1.5 mg) and weekly treated by IA administration of FLL at 100 μg/mL for 4 weeks. Thermal withdrawal latency, mechanical withdrawal threshold, and spontaneous activity were tested for evaluation of pain behavior, and histopathological (HE, SO, and ABH staining) and immunohistochemical (Col2, Col10, and MMP13) analyses were conducted for observation of cartilage degradation. *In vitro* effect of FLL on chondrocytes was evaluated by MTT assay and qPCR analysis. Moreover, HPLC analysis was performed to determine its chemoprofile. The pain behavioral data showed that FLL attenuated joint pain hypersensitivity by increasing thresholds of mechanical allodynia and thermal hyperalgesia as well as spontaneous activity. The histopathological result showed that FLL reversed OA cartilage degradation by protecting chondrocytes and extracellular matrix in cartilage, and the immunohistochemical analysis revealed its molecular actions on protein expressions of MMP13, Col2, and Col10 in cartilage. The MTT assay showed its proliferative effects on chondrocytes, and qPCR assay clarified its mechanism associated with gene expressions of *Mmp13*, *Col2*, *Col10*, *Adamts5*, *Aggrecan*, and *Runx2* in TNF-α treated chondrocytes. Our results revealed an anti-OA effect of FLL on pain behavior and cartilage degradation in OA rats and clarified a molecular mechanism in association with the suppression of chondrocyte hypertrophy and catabolism. IA FLL can be regarded as novel and promising option for OA therapy.

## Introduction

As the most common joint disease worldwide, osteoarthritis (OA) affects an estimated 15–40% populations aged 60 years or older (Zhang and Jordan, 2010; Cross et al., 2014). The prevalence of OA is increasing year by year with rising rates in aged people (Nguyen et al., 2011). It occurs with synovial inflammation, cartilage breakdown and bone remodeling, developing to a syndrome of chronic pain, stiffness and impaired movement. The pain and decreased function associated with OA place a major burden on communities as well as health and social care systems, making OA a leading cause of global disability (Zhang and Jordan, 2010; Wittenauer et al., 2013). The harms of OA to human beings are spreading rapidly, especially in developed countries, where people live longer and the population undergoes aging (Felson et al., 2016). Many efforts have been undertaken to develop anti-OA therapies. However, there is no disease-modifying therapy available for OA to date, resulting in joint replacement as inevitable and eventual approach for patients with end-stage OA (Bijlsma et al., 2011; Carr et al., 2012; Pivec et al., 2012). The arthroplasty surgery always leads to adverse outcomes with high cost, conceivable troubles and finite lifespan of prostheses. Besides, the medical costs of OA account for 1–2.5% of the gross domestic product in developed countries and increase by years with increasing prevalence of OA, leading to large resultant socioeconomic burden (Bitton, 2009). Therefore, the current therapies do not satisfy the clinical needs due to their insufficiency and limitations, urgently demanding a new cost-effective approach to treat OA.

The short-term goal of OA treatment has been set to relieve pain and stiffness to increase function and mobility, and the long-term goal is to stop or slow disease progression to avoid disability and prevent, or at least delay, the need for a total knee replacement (total knee arthroplasty) (Wehling et al., 2017). It is generally accepted that conservative treatment should precede the consideration of surgery (Hunter and Felson, 2006; Wittenauer et al., 2013). The conservative treatment options recommended by national and international guidelines are non-pharmacological interventions and systemic pharmacological therapies (Jordan et al., 2003; Hochberg et al., 2012; McAlindon et al., 2014; Roman-Blas et al., 2016). The pharmacological approaches in clinical practice mainly consist of non-steroidal anti-inflammatory drugs (NSAIDs), analgesics (e.g., acetaminophen and synthetic opioids), hyaluronic acid, and corticosteroids. However, these medications do not meet patients’ expectancy and even cause serious side effects (Richette et al., 2015; Roberts et al., 2015). Traditional Chinese medicine (TCM) has been accepted as promising alternative option for OA therapy, not only in Asian countries but also in the West, which might result from its effects on pain, loss of mobility, and dysfunction (Williamson et al., 2007; Essex et al., 2016; Li et al., 2017; Yang et al., 2017). Currently, the use of TCM has been included in the OARSI (Osteoarthritis Research Society International) guidelines and advocated by reviews for conservative management of OA (McAlindon et al., 2014; Hou et al., 2015).

*Fructus Ligustri Lucidi* (FLL), known as *Nv Zhen Zi* in Chinese, is the dried ripen fruit of *Ligustrum lucidum Ait*. (Oleaceae.). It was originally recorded in the earliest existing Pharmacopeia “*Shennong Ben Cao Jing”* (*Shennong’s Herbal*) during the East Han Dynasty of China (25-220 AD). It has been clinically used for over 1000 years, mainly to treat age-related diseases such as osteoporosis, arthritis, and bone pain (Pang et al., 2015; Chen et al., 2017). In the Chinese Pharmacopeia, FLL is recognized as an edible herb with kidney-tonifying activity and is effective in treating soreness and weakness of waist and knees (China Pharmacopeia Committee, 2015). According to the theory and clinical experiences of TCM, kidney-deficiency is a pattern related to the pathogenesis of bone and joint diseases, and kidney-tonifying activity represents a bone-strengthening effect that facilitates bone metabolism and morphogenesis (Chen et al., 2016). Many herbs with kidney-tonifying activity have been clinically used to treat OA, resulting in positive outcomes (Yuan et al., 2011; Chen et al., 2015). The above implies that FLL may have potential effect against OA and can thereby be postulated as a potential candidate anti-OA agent. However, there is no report on FLL’s anti-OA effect so far. Thus, for the first time, we applied OA model of rats and evaluated the anti-OA effect of FLL extract *in vivo* and *in vitro*.

## Materials and Methods

### Medicinal Materials and FLL Preparation

Dried mature fruits of *Ligustrum lucidum Ait*. (Oleaceae.) were purchased from Zhejiang Chinese Medical University Medicine Yinpian Factory (Hangzhou, China) and authenticated by the authors. A voucher specimen (No.: 17072201) has been deposited at the Institute of Orthopaedics and Traumatology of the First Affiliated Hospital of Zhejiang Chinese Medical University (Hangzhou, China). The raw materials were prepared in accordance with a traditional procedure (China Pharmacopeia Committee, 2015) and powered. Briefly, the powder was mixed with 10-fold destilled water and boiled twice for 30 min each. Then, the liquid supernatant was collected and concentrated by Labconco FreeZone 6plus (Labconco Co., KS, United States), named FLL.

### Chemicals and Reagents

The standard substance of nuezhenide (C_31_H_42_O_17_; MW: 686.62) (99.8% of purity) (batch number: B21240) was purchased from purchased from the National Institute for the Control of Pharmaceutical and Biological Products (Beijing, China). Iscove’s modified Dulbecco’s medium (IMDM), fetal bovine serum (FBS) and 0.25% trypsin were obtained from Gibco (Thermo Fisher Scientific, Inc., Waltham, MA, United States). 3-(4,5-Dimethylthiazol-2-yl)-2,5-diphenyltetrazolium bromide (MTT) and dimethyl sulfoxide (DMSO) were obtained from Sigma-Aldrich (Taufkirchen, Germany). TRIzol reagent was purchased from Thermo Fisher Scientific Inc. The real time polymerase chain reaction (PCR) kit was purchased from Takara Biotechnology Co., Ltd. (Dalian, China). Mono-iodoacetate (MIA) was purchased from Sigma (St. Louis, MO, United States). All antibodies were purchased from Cell Signaling Technology, Inc. (Danvers, MA, United States).

### Chromatographic Analysis

HPLC analysis was performed on an Agilent 1260 Infinity HPLC system (Agilent Technologies, CA, United States). Chromatographic separation was achieved on an Agilent Extend C_18_ column (250 × 4.6 mm, 5 μm) (Shandon Scientific, Cheshire, United Kingdom) at 30°C. The mobile phase consisted of acetonitrile and water (20:80) with a flow rate of 1.0 mL/min. The sample injection volume was 10 μL and the detection wavelength was 224 nm. The standard substance of nuezhenide was dissolved in water at concentrations of 1.25, 2.5, 5, 10, 20, 40, 80 μg/mL and analyzed to obtain the linear regression equation and linear dependent coefficient (*R*^2^). The *R*^2^ was used to evaluate the linearity of variable *X* (concentration of nuezhennide) versus *Y* (peak area), with the value of *R*^2^ decreasing from 1 as the linear fit decreases. Then the content of nuezhenide in the extract of FLL was calculated using the linear regression equation, following three parallel analyses on the FLL sample.

### Animal and Cell Line

Male SD rats weighing 180-220 g were purchased from Shanghai Super B&K Laboratory Animal Co. Ltd. (Grade SPF II, SCXK (Shanghai): 2013-0016) and housed under controlled pathogen-free conditions with a 12 h light/dark cycle and allowed food and water *ad libitum*. All animals were treated in strict accordance with the China legislation on the use and care of laboratory animals and the animal experiment was approved by the Medical Norms and Ethics Committee of Zhejiang Chinese Medical University. Primary chondrocyte was isolated from the rat articular cartilage and cultured as previously described (Tong et al., 2014).

### Animal Experiment

Thirty animals were randomly and equally divided into three groups: NC as normal control group, Model as OA group, and OA+FLL as FLL treated OA group. Rats in the NC group were intra-articularly injected with 50 μL of saline, while all the other groups were treated with 50 μL of 30 mg/mL MIA to establish the OA model. Seven days later, the FLL groups were weekly treated with 50 μL of FLL at 100 μg/mL by intra-articular injection, while the NC group was given an equal volume of distilled water. The treatment was lasted for 4 weeks, and the behavior parameters TWL (thermal withdrawal latency), MWT (mechanical withdrawal threshold), and spontaneous activity were tested after the final treatment. At the end, all animals were sacrificed under anesthesia and their knee joints were immediately taken for histopathological and immunohistochemical analyses.

### Behavioral Assessments

The MWT and TWL were measured by the von Frey test (Chaplan et al., 1994) and a Plantar Test apparatus (UgoBasile, Italy), respectively. All rats were placed in elevated plastic cages with wire mesh bases suspended above a table and given 30 min for acclimatization to the testing environment. The von Frey needle was pressed perpendicularly against the plantar surface of the left and right hind paws of each rat for more than three times. For testing the thermal hyperalgesia, a focused beam of radiant heat (up to 35°C) was irradiated to the plantar surface of the hind paws for more than three times and held for maximal 20 s. A positive response for each test was regarded as the sharp withdrawal of the paw and paw licking. The spontaneous activity test was conducted using an animal behavior analysis system with a spontaneous activity video analysis system. Each rat was placed in every spontaneous activity box and the activity number recorded for 10 min. The box was kept in dark, and the environment was quiet.

### Histopathological and Immunohistochemical Analyses

The joints of all rats were resected, fixed in 10% formalin for 24 h, decalcificated with 10% EDTA in PBS for 8 weeks, and embedded in paraffin. Sections (2–3 μm) were stained with HE (hematoxylin and eosin), SO (safranin-O) and ABH (alcian blue/hematoxylin) as well as antibodies against rat type II collagen (Col2), type X collagen (Col10) and matrix metalloproteinase 13 (MMP13). All slides were examined under microscopy and were statistically graded on a scale of 0-13 by double-blind observation, according to Mankin’s scoring system (Mankin et al., 1971).

### Cell Viability Assay

The chondrocyte viability was determined by MTT assay. Cells were seeded on 96-well plates at a density of 5 × 10^3^ cells/well in 200 μL medium for 24 h, followed by the treatment of FLL at 0, 5, 10, 15, 25, 50, 100, 200 μg/mL for 24 and 48 h. A total of 20 μL MTT solution (5.0 mg/mL) was added to each well and incubated at 37°C for 4 h. 150 μL DMSO was subsequently added to each well to dissolve the formazan crystals and the optical density (OD) value was measured at 490 nm with a microplate reader (Bio-Rad Laboratories, Inc., Hercules, CA, United States). Proliferative rate (PR, %) = (EAA-treated OD/untreated OD) × 100.

### qPCR (Real Time PCR) Analysis

Chondrocytes were pre-treated with MIA (0.5 μg/mL) and TNF-α (10 ng/mL), respectively, for 6 h, and then treated with EAA (80 mg/mL) for 24 h. The relative mRNA expression of targeted genes in chondrocytes was measured using a qPCR assay on an ABI QuantStudio^TM^ 7 Flex Real-Time PCR System (Applied Biosystems; Thermo Fisher Scientific, Inc.). Total RNA was extracted from chondrocytes using TRIzol reagent and reverse transcription was performed to produce cDNA. The final PCR reaction system was 20.0 μL, comprising 10.0 μL SYBR^®^ Premix Ex Taq II (Tli RnaseH Plus), 0.8 μL PCR forward primer, 0.8 μL PCR reverse primer, 2.0 μL template cDNA, 0.4 μL ROX reference dye and 6.0 μL ddH_2_O. The qPCR reaction conditions were as follows: 95°C for 30 s for initial denaturation, followed by 40 cycles of denaturation at 95°C for 5 s, annealing at 60°C for 34 s and extension at 72°C for 40 s. At the end of each reaction, melting curve analysis was performed. *β-actin* was used as the reference gene and the 2^-ΔΔCT^ method was used to analyze the relative mRNA expressions (Table 1).

**Table 1:** Primer sequences used for qPCR analysis.

Gene	Forward primer	Reverse primer
*β-actin*	5′-CCCGCGAGTACAACCTTCT-3′	5′-CGTCATCCATGGCGAACT-3′
*Col2*	5′-CTCAAGTCGCTGAACAACCA-3′	5′-GTCTCCGCTCTTCCACTCTG-3′
*Col10*	5′-GATCATGGAGCTCACGGAAAA-3′	5′-CCGTTCGATTCCGCATTG-3′
*Mmp13*	5′-CTATGGTCCAGGAGATGAAGAC-3′	5′-GTGCAGACGCCAGAAGAATCT-3′
*Adamts5*	5′-TGGAGTGTGTGGAGGGGATA-3′	5′-CGGACTTTTATGTGGGTTGC-3′
*Aggrecan*	5′-GCAGACATTGATGAGTGCCTC-3′	5′-CTCACACAGGTCCCCTCTGT-3′
*Runx2*	5′-CCATAACGGTCTTCACAAATCCT-3′	5′-RTCTGTCTGTGCCTTCTTGGTTC-3′


### Statistical Analysis

Data are expressed as mean ± SD. Data were analyzed using one-way ANOVA followed by Fisher’s least significant difference (LSD) comparison. All analyses were performed using an updated version of DPS software (Tang and Feng, 2007).

## Results

### Chemoprofile of FLL

**FIGURE 1:**
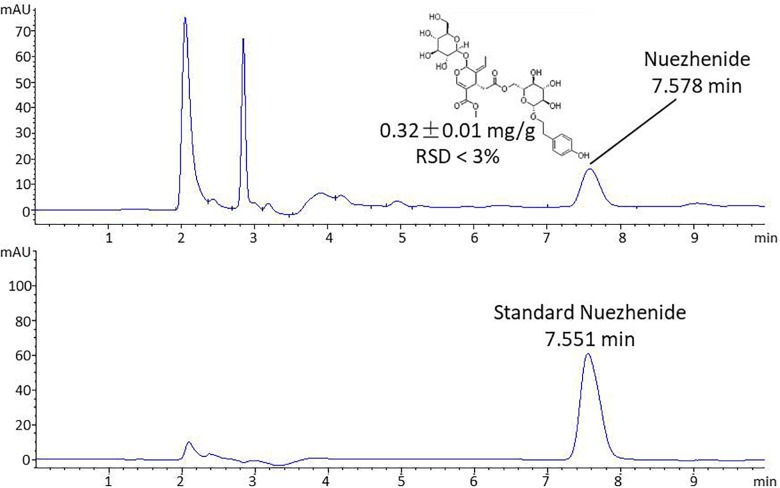
HPCL chromatogram of FLL and content of nuezhenide.

The HPLC chromatographic profile of FLL is shown in Figure 1. The linear regression equation was obtained: *Y* = 6.36 *X* – 4.9449, *R*^2^ = 0.9995, indicating a good linear relationship in the concentration range of 1.25–80 μg/mL of nuezhenide. The extract of FLL contained nuezhenide with a peak at 7.578 min of retention time. The content of nuezhenide in the FLL extract was calculated as 0.32 ± 0.01 mg/g with RSD % of 2.28 (*n* = 3).

### Pain-Relieving Effect of FLL

**FIGURE 2:**
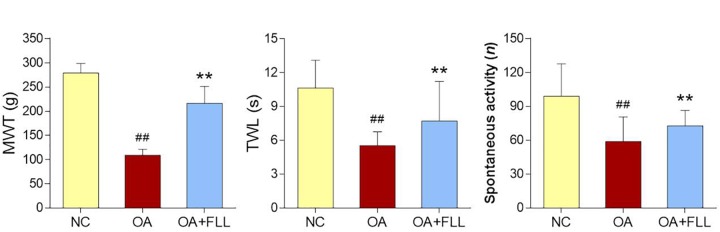
MWT (g), TWL (s), and spontaneous activity (n) results of rats after final FLL treatment (28 days). Values are presented as mean ± SD. ^##^*P* < 0.01 versus NC group on day 28; ^∗∗^*P* < 0.01 versus OA model group on day 28.

Pain related behavioral responses are shown in Figure 2, in which MWT and TWL reflect mechanical allodynia and thermal hyperalgesia, respectively. After the OA modeling, MWT, TWL, and spontaneous activity were significantly decreased in comparison with the normal control (all *P* < 0.01). As compared to the model, FLL significantly increased the levels of MWT, TWL, and spontaneous activity after 28 days intra-articular treatment (all *P* < 0.01).

### *In vivo* Anti-OA Effect of FLL

**FIGURE 3:**
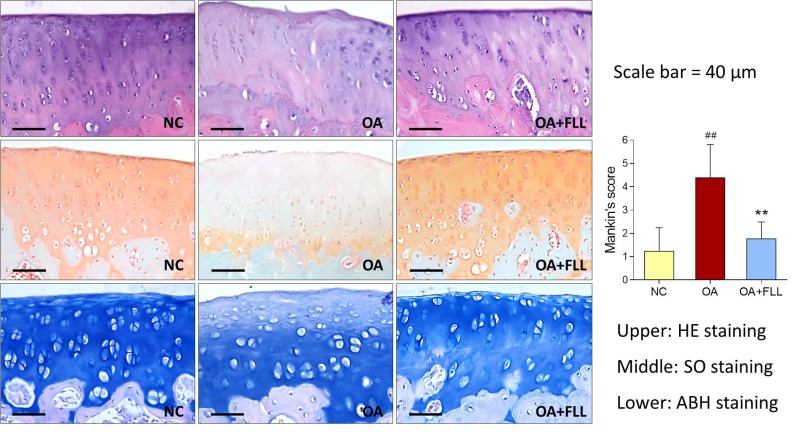
Histopathological observation (HE, SO, and ABH staining) and Mankin’s scoring of rat knee joints at day 28 after FLL treatment. Values are presented as mean ± SD. ^##^*P* < 0.01 versus NC group; ^∗∗^*P* < 0.01 versus OA model group.

Histopathological results with HE, SO, and ABH staining are shown in Figure 3. Obvious cartilage degeneration, characterized by chondrocyte loss, collagen disruption, matrix disorganization, and cartilage surface irregularity, was observed in the OA model group with significant increase of Mankin’s score (*P* < 0.01 versus NC). The degenerative phenotype was reversed by intra-articular FLL treatment, with significant decrease of Mankin’s score (*P* < 0.01 versus OA model). The number and arrangement of chondrocyte, mass of collagen, and cartilage surface were obviously improved by FLL.

**FIGURE 4:**
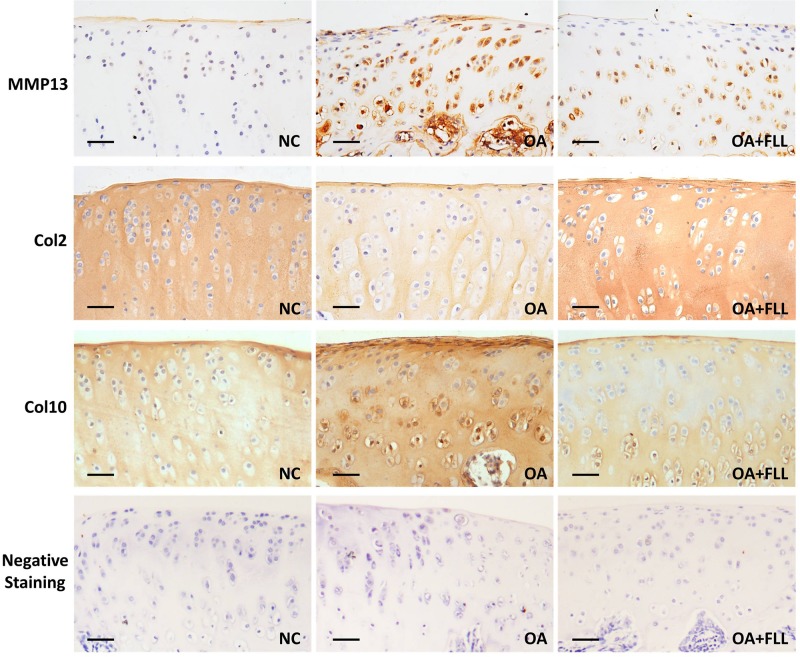
Immunohistochemical observation of the expressions of MMP13, Col2, and Col10 in rat cartilage at day 28. Negative staining was used as negative control that reacted with no primary antibodies but only secondary antibodies. Scale bar = 50 μm.

Immunohistochemical results using anti-MMP13, anti-Col2, and anti-Col10 antibodies are shown in Figure 4. The normal control cartilage showed negative reaction with anti-MMP13 antibody, while the OA model cartilage showed strongly positive reaction with this antibody, indicating increased MMP13 expression in the cartilage of OA rats. With FLL treatment, the MMP13 expression on cartilage was remarkably reduced in OA+FLL group. A similar trend was found with anti-Col10 antibody that the cartilage of OA group overexpressed larger amount of Col10 than that of normal control group and OA+FLL group. Moreover, cartilage of OA group expressed a lesser amount of Col2 than that of normal group, while FLL restored the abnormal Col2 expression to normal level in OA+FLL group.

### Cellular and Molecular Actions of FLL on Chondrocytes

**FIGURE 5:**
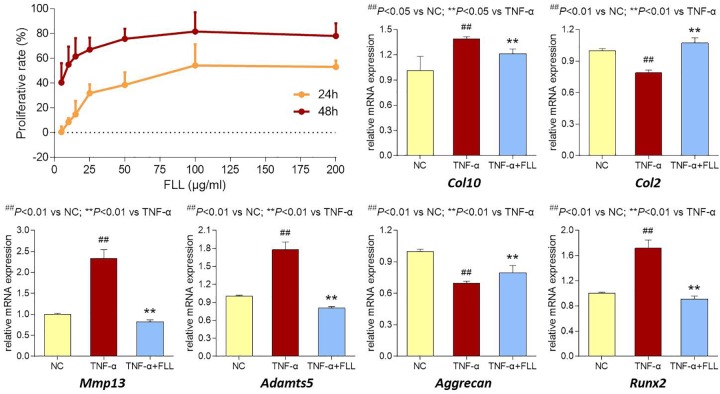
Cell viability of chondrocytes with FLL treatment (upper left) for 24 and 48 h. Relative mRNA expressions of OA-related genes in chondrocytes treated with TNF-α or TNF-α plus FLL for 24 h. Values are presented as mean ± SD.

The proliferative effect of FLL on chondrocytes was assessed by MTT assay. As shown in Figure 5 (upper left), FLL at a dose range of 5–200 μg/mL significantly increased the cell viability of chondrocytes with increasing proliferative rate from 0.6 to 53.1% after 24 h treatment and from 40.5 to 78.0% after 48 h treatment. The proliferative effect of FLL on chondrocytes was in a dose-dependent manner.

The modulation effects of FLL on the expression of OA-related genes in chondrocytes were determined using qPCR assay. Chondrocytes were pretreated with TNF-α to mimic OA pathological condition. As shown in Figure 5 (upper right and lower), TNF-α significantly upregulated the expressions of *Col10*, *MMP13*, *Adamts5*, and *Runx2* and downregulated the expressions of *Col2* and *Aggrecan*, as compared with that of normal control (all *P* < 0.05 or 0.01). Following 24 h treatment of FLL, the abnormal expressions of those genes were significantly reversed toward the normal levels, as compared with that of TNF-α group (all *P* < 0.05 or 0.01).

## Discussion

According to the TCM theory, FLL is an herb with bitter and sweet “flavor” as well as cool “property” (China Pharmacopeia Committee, 2015). The ancient terms “flavor” and “property” represent the physiological responses of patients to TCM herbs. Moreover, FLL is distributed in the kidney meridian, indicating its target-specific reaction with bone. Bone and joint diseases, such as osteoarthritis and osteoporosis, are defined as “bone atrophy,” “bone loss,” or “bone rheumatism” in TCM, being attributed to the TCM syndrome of kidney-deficiency (Guo et al., 2015). Meanwhile, FLL exerts bone-protective effect due to its kidney-tonifying activity against kidney deficiency, and has thereby been clinically used to treat osteoporosis and bone metabolic disorders (Chen et al., 2017). Previous studies showed that FLL extract modulated the turnover of bone and the calcium balance in ovariectomized (OVX) rats (Zhang et al., 2006). It was able to suppress an increased level of osteocalcin (OCN) in serum and deoxypyridinoline (DPD) in urine, and improve calcium metabolism by increasing intestinal calcium absorption, and inhibiting urinary calcium excretion in OVX rats (Zhang et al., 2006; Chen et al., 2017). Furthermore, FLL was found to improve bone properties in rats by increasing bone mineral density (BMD) and biomechanical strength in the tibia diaphysis, improving bone mineral content (BMC) at both tibia and femoral diaphysis and lumbar vertebra, and inhibiting bone loss of cortical and trabecular bone (Zhang et al., 2008a,b). Thus, FLL was postulated as a potential candidate in the treatment of bone and joint diseases.

Our preliminary study evaluated three doses (50, 100, 200 μg/mL) of FLL and found that 50 μg/mL of FLL showed no effect and 200 μg/mL of FLL exerted a similar effect to that of 100 μg/mL of FLL, indicating no dose-dependent manner of FLL (Supplementary Figure S1). In this study, the anti-OA effect of FLL at 100 μg/mL was evaluated by *in vivo* and *in vitro* experiments. The animal experiment showed that FLL attenuated joint pain hypersensitivity by increasing thresholds of mechanical allodynia and thermal hyperalgesia as well as spontaneous activity (Figure 2). Moreover, FLL reversed OA cartilage degradation by protecting chondrocytes and extracellular matrix (ECM) in cartilage (Figure 3). The immunohistochemical analysis revealed FLL’s underlying mechanism associated with the regulations of MMP13, Col2, and Col10 expressions in cartilage (Figure 4). qPCR assay further clarified the mechanism that FLL not only reversed the abnormal expressions of *Mmp13*, *Col2*, and *Col10* but also restored the expressions of *Adamts5*, *Aggrecan*, and *Runx2* in TNF-α treated chondrocytes (Figure 5). Articular cartilage is composed of chondrocytes embedded in ECM, which provides the bioactive and biomechanical characteristics that are essential for articular movement (Huang and Wu, 2008). Col2 and aggrecan are the major ECM components of cartilage, and loss of them is the sine qua non of OA. As an early marker of chondrogenesis, Col2 represents 90–95% of its total collagen content and forms the fibrils that give cartilage its tensile strength. Cleavage of Col2 by collagenases is excessive in OA cartilage (Billinghurst et al., 1997). Besides, aggrecan in articular cartilage is also an early pathophysiological marker for OA, since it is largely responsible for the high resistance of ECM to compression of the load-bearing tissue (Caterson et al., 2000; Arner, 2002; Bondeson et al., 2008). MMP13 is a major matrix metalloproteinase/collagenase targeting cartilage for OA degradation. It degrades Col2 and aggrecan in cartilage during OA progression (Shiomi et al., 2010). Preclinical studies showed that *Mmp13*-overexpressing transgenic mice developed a spontaneous OA-like articular cartilage destruction phenotype, whereas *Mmp13*-knockout transgenic mice decelerated OA progression by decreasing articular cartilage degeneration after surgical OA modeling (Neuhold et al., 2001; Wang et al., 2013). In addition, clinical data revealed that OA patients have high MMP13 expression in chondrocytes (Roach et al., 2005). Aggrecanase, such as ADAMTS-5, is overexpressed in osteoarthritic articular chondrocytes, which changes the physiological balance between matrix synthesis and degradation and results in an enhanced aggrecan proteolysis (Song et al., 2007; Echtermeyer et al., 2009). Gene deletion of ADAMTS-5 has shown protection against cartilage degradation in a surgical model of OA (Glasson et al., 2005; Stanton et al., 2005). Runx2 (Runt-related transcription factor 2) is an osteogenic transcriptional activator, functioning in chondrocyte hypertrophy and osteophyte formation in the pathogenesis of OA (Kamekura et al., 2012). It binds to the DNA sequence (osteoblast-specific element 2, OSE2) which constitutes the proximal promoter of *Mmp13* gene (Mengshol et al., 2001). Overexpression of Runx2 activates matrix degradation (MMP13 and ADAMTS5) through direct regulation of *Mmp13* gene transcription (Wang et al., 2012). Chondrocytes always undergo hypertrophy before suffering apoptosis in the process of OA. Col10 is a late-stage chondrocyte hypertrophy marker upregulated in OA cartilage as a result of chondrocyte hypertrophy and cartilage calcification (von der Mark et al., 1992; Walker et al., 1995). Clinically, it is associated with cartilage degradation and inflammation in patients with various degrees of OA (He et al., 2014). The upregulation of Col10 has been reported in surgical animal OA model as well (Kamekura et al., 2005). In this study, along with a typical OA phenotype and genotype, expressions of the above molecules were abnormally regulated by MIA, which were eventually reversed by FLL.

We applied intra-articular (IA) injection as a drug delivery system for FLL treatment on OA. Localized IA therapy delivered directly into the affected joint is a representative of conservative therapies for OA (Wittenauer et al., 2013). Because OA lesions are only present in joints, local treatment administered through the IA route is an appropriate strategy. In 1951, Hollander and colleagues introduced IA treatment in arthritic joints for the first time (Hollander et al., 1951), and later on IA therapies were approved by Food and Drug Administration (FDA) and European Medicines Agency (EMA). Compared to the conventional oral administration, this technique avoids systemic exposure and potential adverse side effects (Nguyen and Rannou, 2017). Indeed, IA injection has many intrinsic features, which might provide advantages over systemic therapies: increased bioavailability and safety, lower drug dose and systemic toxicity, avoidable conventional barriers to joint entry, and a positive placebo benefit (Wehling et al., 2017). In addition, it is a relatively simple and well tolerated procedure associated with minimal recovery time, which can be used as an attractive alternative modality for delivering drugs with low oral bioavailability (Wehling et al., 2017; Maudens et al., 2018). Clinical trials provided evidence that IA therapies represent an alternative and safe treatment in OA patients with lower rate of withdrawals related to adverse events than conventional therapies (Bannuru et al., 2015; Nguyen et al., 2016). Various studies have conducted comparisons of the cost-effectiveness between IA therapies and conventional ones, revealing lesser costs per quality-adjusted life years gained with IA therapies (5785–9039 US dollars) versus that of conventional ones (10,716 US dollars) (Losina et al., 2013; Rosen et al., 2016; Belzile et al., 2017). IA injection is an innovative way that may contribute to the effectiveness and safety of TCM therapies. This study is our first attempt to combine an IA injection and TCM treatment, providing an alternative treatment option to complement the traditional strategies. Oral administration is the traditional route for TCM treatment. However, knowledge of the differences between IA injection and oral administration is still limited in the TCM field, and larger and longer-term studies are therefore needed to obtain more positive results and further substantiate the applicability of IA TCM therapies.

## Conclusion

In conclusion, our data demonstrated that FLL effectively ameliorated pain response and protected chondrocytes and ECM from degradation in OA rats. FLL exerted the effect by increasing the resistance of cartilage to MIA-induced damage and preventing chondrocytes from TNF-α induced hypertrophy. The mechanism of action is associated with the regulations of protein expressions of MMP13, Col2, and Col10 in cartilage and gene expressions of *Mmp13*, *Col2*, *Col10*, *Adamts5*, *Aggrecan*, and *Runx2* in chondrocytes. Although more studies are needed to further clarify the underlying mechanism of FLL against OA, our data provide certain knowledge of its anti-OA effect and may place FLL as novel and potential therapeutic choice for OA therapy in the future.

## Author Contributions

BY, LZ, and CW performed the main experiments of this study. LYa, LYu, FL, WD, and GY contributed to the materials acquisition and data analysis for this study. LS designed the main part of this work and drafted the manuscript. PT and QY improved the design and provided funding support to this work. TE improved the design, draft, and language editing of this manuscript. All listed authors approved the manuscript for publication and agreed to be accountable for all aspects of this work.

## Conflict of Interest Statement

The authors declare that the research was conducted in the absence of any commercial or financial relationships that could be construed as a potential conflict of interest.

## Supplementary Material

Figure S1 Histopathological observation with HE staining and Mankin’s scoring of rat knee joints at day 28 after FLL treatment at 50, 100, and 200 μg/mL. Values are presented as mean ± SD. ^##^*P* < 0.01 vs. NC group; ^∗∗^*P* < 0.01 vs. OA model group.
