# Supplementary figures and images for: Intra-Articular Injection of Fructus Ligustri Lucidi Extract Attenuates Pain Behavior and Cartilage Degeneration in Mono-Iodoacetate Induced Osteoarthritic Rats
Source: Front Pharmacol. 2018 Nov 23;9:1360. doi: 10.3389/fphar.2018.01360 (PMC6265644; doi:10.3389/fphar.2018.01360)

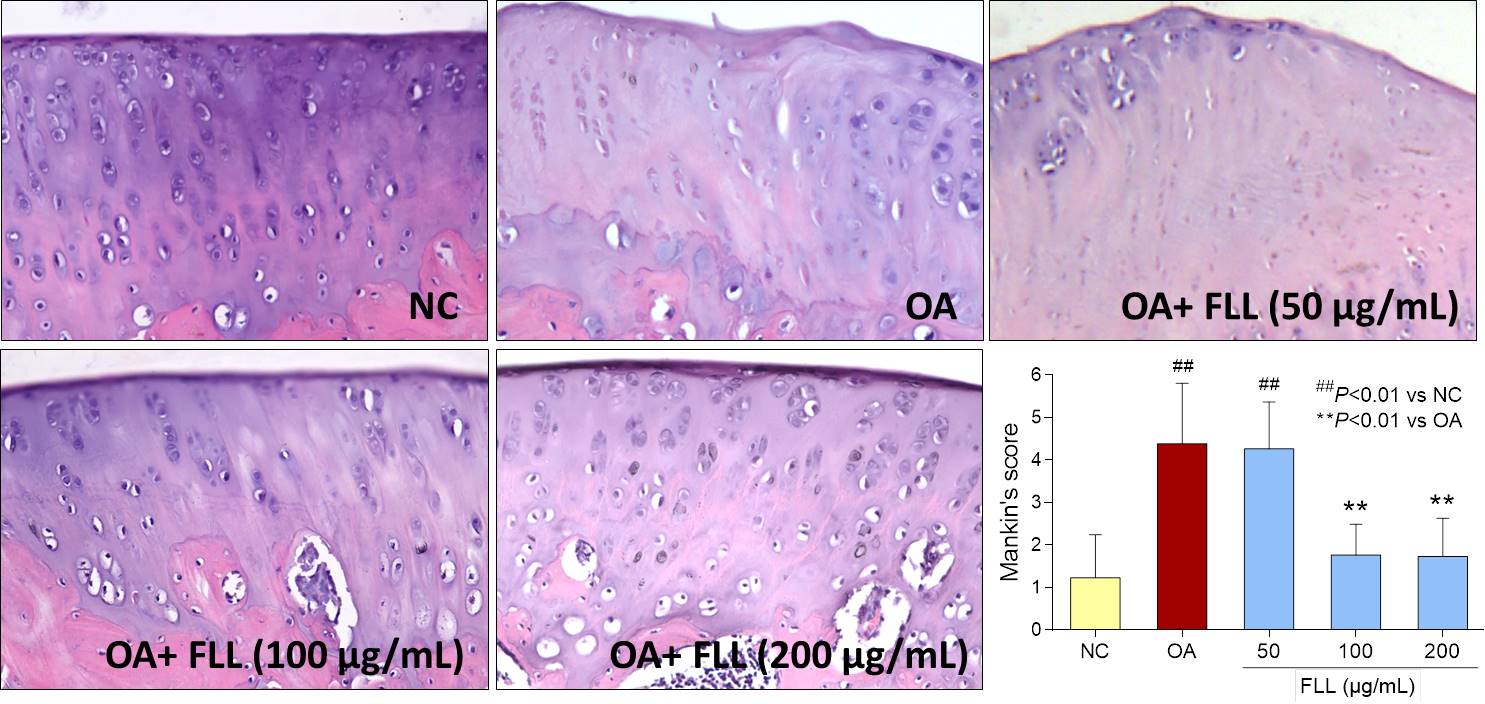

Supplement: Figure S1 — Histopathological observation with HE staining and Mankin’s scoring of rat knee joints at day 28 after FLL treatment at 50, 100, and 200 μg/mL. Values are presented as mean ± SD. ##P < 0.01 vs. NC group; ∗∗P < 0.01 vs. OA model group. [file Image_1.JPEG]
